# TFA Generation and Deposition over Europe May Currently See a Greater Influence from HFO-1234yf than HFC-134a

**DOI:** 10.1021/acs.estlett.6c00356

**Published:** 2026-06-15

**Authors:** Rayne Holland, Ben Adam, M Anwar H Khan, Dickon Young, Simon O’Doherty, Kieran M. Stanley, Matthew Rigby, Dudley E. Shallcross

**Affiliations:** † School of Chemistry, 1980 University of Bristol, Cantock’s Close, Bristol BS8 1TS, U.K.; ‡ Department of Chemistry, University of the Western Cape, Robert Sobukwe Road, Bellville 7535, South Africa

**Keywords:** TFA, trifluoroacetic acid, HFO-1234yf, HFC-134a, deposition, global burden

## Abstract

Concerns around the prevalence of trifluoroacetic acid (TFA, CF_3_COOH) in the environment have been ongoing for more than 20 years. One source of TFA to the environment is via the atmospheric degradation of fluorinated gases (F-gases) to TFA and its subsequent deposition. During the past decade, controls on one major class of F-gases, hydrofluorocarbons (HFCs), have resulted in an increase in the production and use of hydrofluoroolefins (HFOs). Here, a tropospheric chemical transport model, STOCHEM-CRI, is used to compare the amount of TFA generated by the breakdown of an abundant HFC, HFC-134a (1,1,1,2-tetrafluoroethane, CF_3_CH_2_F), and its typical replacement, HFO-1234yf (2,3,3,3-tetrafluoropropene, CH_2_=CFCF_3_). Using derived global emission estimates for 2023, we predict that HFO-1234yf is already dominating atmospheric generation and deposition of TFA over Europe when compared with HFC-134a. This is despite the fact that the derived global emissions of HFC-134a are 22 times higher than those of HFO-1234yf. Globally, our simulations show that TFA production from HFO-1234yf is already equivalent to 26–75% of the TFA production from HFC-134a. As the transition away from HFCs progresses, it is likely that HFO-1234yf will continue to increase in its global contribution to TFA generation and resultant environmental contamination.

## Graphical abstract

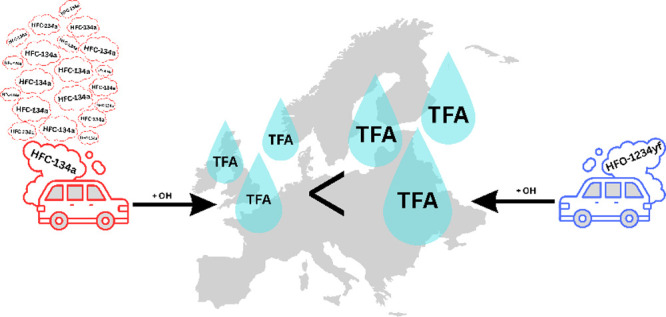

## Introduction

Trifluoroacetic acid (TFA, CF_3_COOH) is a persistent organic pollutant receiving considerable attention in the scientific community, and more widely, due to its environmental persistence and designation as a “forever chemical”.^1–5^ While there is little evidence that TFA is toxic at levels currently observed in the environment, published ecotoxicity data are limited and concentrations are reported to be increasing in some plant matter^6^ and environmental aqueous phases (including drinking water).^3,7–11^ TFA has also been detected in human blood and urine.^12–14^

A significant anthropogenic source of TFA to the atmosphere, and wider environment, is expected to be via atmospheric degradation of compounds that have replaced chlorofluorocarbons (CFCs) in applications such as refrigeration and foam blowing. These include hydrofluorocarbons/hydrochlorofluorocarbons (HFCs/HCFCs)^5,15–19^ and hydrofluoroolefins (HFOs),^20,21^ many of which generate TFA to varying extents.^22^

CFCs were replaced by HCFCs and then HFCs, due to the controls of the Montreal Protocol,^23,24^ with each class of compounds having lower ozone depletion potentials (ODPs) than their predecessors. However, many commonly used HFCs have high global warming potentials (GWPs), and concerns over their future impact on global radiative forcing resulted in the Kigali amendment to the Montreal Protocol, which mandated their phase-out.^25^ It is anticipated that the primary replacement for HFCs will be HFOs due to their physicochemical similarity and their ability to be used in current infrastructure. HFOs typically have low ODPs and GWPs^26,27^ due to their reactive olefinic bonds and therefore short atmospheric lifetimes (typically days to weeks^27^), making them a favorable alternative to previous generations of F-gases with respect to climate impacts. However, concerns have been raised around their degradation products, which can form compounds with high GWPs (e.g., HFC-23 and CF_4_)^28,29^ and persistent pollutants like TFA.

Certain HFCs and many HFOs generate TFA via OH-oxidation in the atmosphere. However, due to the elevated reactivity of HFOs, they generally produce TFA at higher yields and at much faster rates than HFCs.^22,30^ Given the high solubility of TFA,^31,32^ it is rapidly and primarily lost from the atmosphere by wet deposition.^33^ This combination means that HFO use is likely to result in higher, more localized TFA generation, and subsequent deposition, near emission sources when compared with HFC use.^20,21,34^ As a result, it is expected that a transition from HFC to HFO use will both increase the global TFA burden due to the increased yield of TFA from HFOs and impact the spatial distribution of this burden.^34–36^

Past studies primarily focused on a specific HFC-HFO transition: that of HFC-134a (1,1,1,2-tetrafluoroethane, CF_3_CH_2_F) to HFO-1234yf (2,3,3,3-tetrafluoropropene, CH_2_=CFCF_3_).^36–38^ Given the widespread usage of HFC-134a in mobile air-conditioning units and the use of HFO-1234yf as a like-for-like replacement,^39,40^ this remains a good proxy for the wider impact of potential HFC-to-HFO transitions. However, previous investigations have been limited by the poor availability of HFO emission data and uncertainty in the TFA yields of HFCs and HFOs.^34,36^

Here, we update previous work^36^ to incorporate (a) uncertainty in TFA yield estimates for HFC-134a, (b) updated HFC-134a emissions and background mole fraction distributions, and (c) reported HFO-1234yf emission estimates from Northwest Europe. These updates are assimilated into a global chemical transport model, STOCHEM-CRI, to investigate whether HFO-1234yf could dominate over HFC-134a with respect to global and regional TFA generation and deposition.

## Materials and Methods

A global three-dimensional chemical transport model, STOCHEM-CRI, was used to simulate the emissions and atmospheric degradation of HFC-134a and HFO-1234yf to ascertain the impact on TFA generation and deposition. Unless otherwise stated, the model setup is as described in Holland et al.,^36^ and full simulation details can be found in Table 1.

**Table 1:** Descriptions of the Three Modeling Simulations Where HFO-1234yf Is the Simulation with HFO-1234yf as the Source Gas (Generating TFA at a Yield of 100%) and HFC-134a_HY and HFC-134a_LY Are Simulations with HFC-134a as the Source Gas (Generating TFA at Yields of 20% and 7%, Respectively)

	Simulation		
	HFO-1234yf	HFC-134a_HY	HFC-134a_LY
F-gas Global Emission/Gg yr^–1^	12^a^	264.1^b^	
HFC-134a Background Mole Fraction (NH/SH)/ppt	N/A	135.03/123.97^b^	
k_OH_/cm^3^ molecule^–1^ s^–1^	1.26 × 10^–12^ exp(−35/T)^c^	1.03 × 10^–12^ exp(−1620/T)^d^	
k_O3_/cm^3^ molecule^–1^ s^–1^	2.77 × 10^–2 ^ ^e^	N/A	
TFA yield (OH-mediated oxidation)/%	100^f^	20^g^	7^g^
TFA yield (O_3_-mediated oxidation)/%	99.88^h^	N/A	

a Derived from a Northwest European emission estimate reported by Vollmer et al.^41^ (1.5 Gg yr^–1^ for 2023) upscaled to a global estimate according to national GDP totals as described in Adam et al.^42^ to give a global estimate of 12 Gg yr^–1^. This is within the range of low- and high-emission scenarios reported by Hart et al.^43^

b HFC-134a global emissions and hemispheric background mole fractions are taken from Western et al.^44^ for the year 2023.

c From Papadimitriou et al.^26^

d From Burkholder et al.^45^

e From Nielsen et al.^46^

f From Hurley et al.^47^

g From Wallington et al.^15^

h Assumed yield based on Garavagno et al.^29^ report that 0.12% of HFO-1234yf O_3_-mediated oxidation forms CF_4_, leaving 100 – 0.12 = 99.88% to potentially contribute to TFA formation.

For HFC-134a, global emissions are distributed according to the EDGAR v8 inventory (Figure S1).^48^ For HFO-1234yf, derived global emissions were distributed according to the method used previously (Figure S2).^28,36^ This method takes the HFC-134a emissions distributions from 2010 extracted from EDGAR v4.2 in combination with the emission distribution over China extracted from Su et al.^49^ As HFO-1234yf is largely being used as a replacement for HFC-134a (primarily in mobile refrigeration) and this transition is underway,^50^ it follows that HFO-1234yf emissions are likely to be distributed similarly to HFC-134a emissions of the recent past, hence the use of EDGAR v4.2 as in our previous study.^36^ Therefore, and in the absence of any reported global emission distributions for HFO-1234yf, we believe this approach is a satisfactory approximation.

Given the uncertainty in the TFA yield of HFC-134a, two scenarios were considered in this study. The first, HFC-134a_HY (high yield), assumes an upper limit TFA yield of HFC-134a of 20%, while a second, HFC-134a_LY (low yield), uses the reported lower limit of 7%.

An additional loss route, oxidation via ozonolysis, was included for HFO-1234yf for completeness, although this has previously been shown to have a minor contribution to the atmospheric fate of HFO-1234yf.^29^

## Results

When compared with measured atmospheric mixing ratios, modeled concentrations of HFO-1234yf show good agreement, validating our implemented emission values and distributions (Figure S3). The HFC-134a agreement is poorer (mean of 3.4% underprediction across sites) than that for HFO-1234yf, as the increasing trend seen in the measurements is not replicated by the model. As emissions are held constant in the model, increasing trends are unlikely to be replicated. Additionally, it is likely that chemical loss of HFC-134a is being overestimated (modeled lifetime of 9 years as opposed to reported lifetime of 14 years^27^). These two in combination are of little consequence to the outcome of the study as the difference between the measured and modeled concentrations is minimal. Moreover, any overprediction of HFC-134a chemical loss further supports the fact that the estimates presented here, regarding HFO-1234yf contribution to TFA generation and deposition, are conservative.

The modeled lifetime of HFO-1234yf of 11.8 days is in excellent agreement with the literature value of 12 days.^51^ The modeled TFA lifetimes of 3.3 and 4.2 days, for the HFC-134a scenarios and the HFO-1234yf scenario, respectively (with disparity driven by spatial variation in TFA generation), are in reasonable agreement with the total reported lifetime of 5 days.^27^ Comparison between our modeled deposition fluxes and those reported in the literature are presented and discussed in the Supporting Information (Figures S4 and S5 and Text S1).

Our model predicts that, using 2023 emission estimates, HFC-134a generates 18.9–53.9 Gg yr^–1^ of TFA in the troposphere, for the low and high yield scenarios, respectively. This is elevated when compared with that reported previously^43^ (13.8 Gg yr^–1^ when TFA yield is modeled at 9%) though this is expected to be primarily due to variation in emissions and background mole fractions utilized. Comparatively, HFO-1234yf is predicted to generate 11.1 Gg yr^–1^ of TFA. It follows that global generation of TFA from HFO-1234yf is already substantial compared with HFC-134a, with HFO-1234yf generating 26–75% the amount of tropospheric TFA generated by HFC-134a despite derived emissions being 22 times lower.

The HFO-1234yf scenario sees higher maximum TFA surface concentrations than those of either of the HFC-134a scenarios. For all scenarios, peak TFA surface concentrations are seen in the northern hemisphere, and for HFO-1234yf, they are in locations with relatively high emissions, according to the implemented emission distribution (Figure S2). Peak TFA surface concentrations are 1.9 times higher in the HFO-1234yf scenario than in HFC-134a_HY and 6 times higher than in HFC-134a_LY. This is due to the longer lifetime of HFC-134a, which allows it to be transported further from its emission source before breaking down to form the TFA. The peak in the HFO-1234yf scenario is seen over continental Europe (a significant emitter of HFO-1234yf, Figure S2) as opposed to the HFC-134a scenarios, which see their maxima closer to the equator, due to its longer lifetime and higher abundances of OH in this region, in agreement with previous studies.^16,17^

Significantly elevated surface concentrations of TFA are seen over large parts of Europe, parts of the United States, and East Asia (Figure 1a,c). Given that we derive global emissions of HFO-1234yf from reported emissions for Europe and scale according to a simple GDP weighting, estimations outside this region (where emissions are extrapolated) are likely more uncertain. The peaks seen in North America and East Asia are a product of the emission distribution imposed in the model, which prescribes high emissions to these areas. However, if emissions in these areas are similar to or exceed those in Europe,^52^ we could reasonably expect TFA deposition from HFO-1234yf to similarly dominate in these areas. We suggest that the results presented here highlight the need for better constraint of global emission distributions of HFO-1234yf with a specific focus on regional emissions in North America and East Asia, especially considering HFO use in both areas is projected to increase.^53,54^

**Figure 1:**
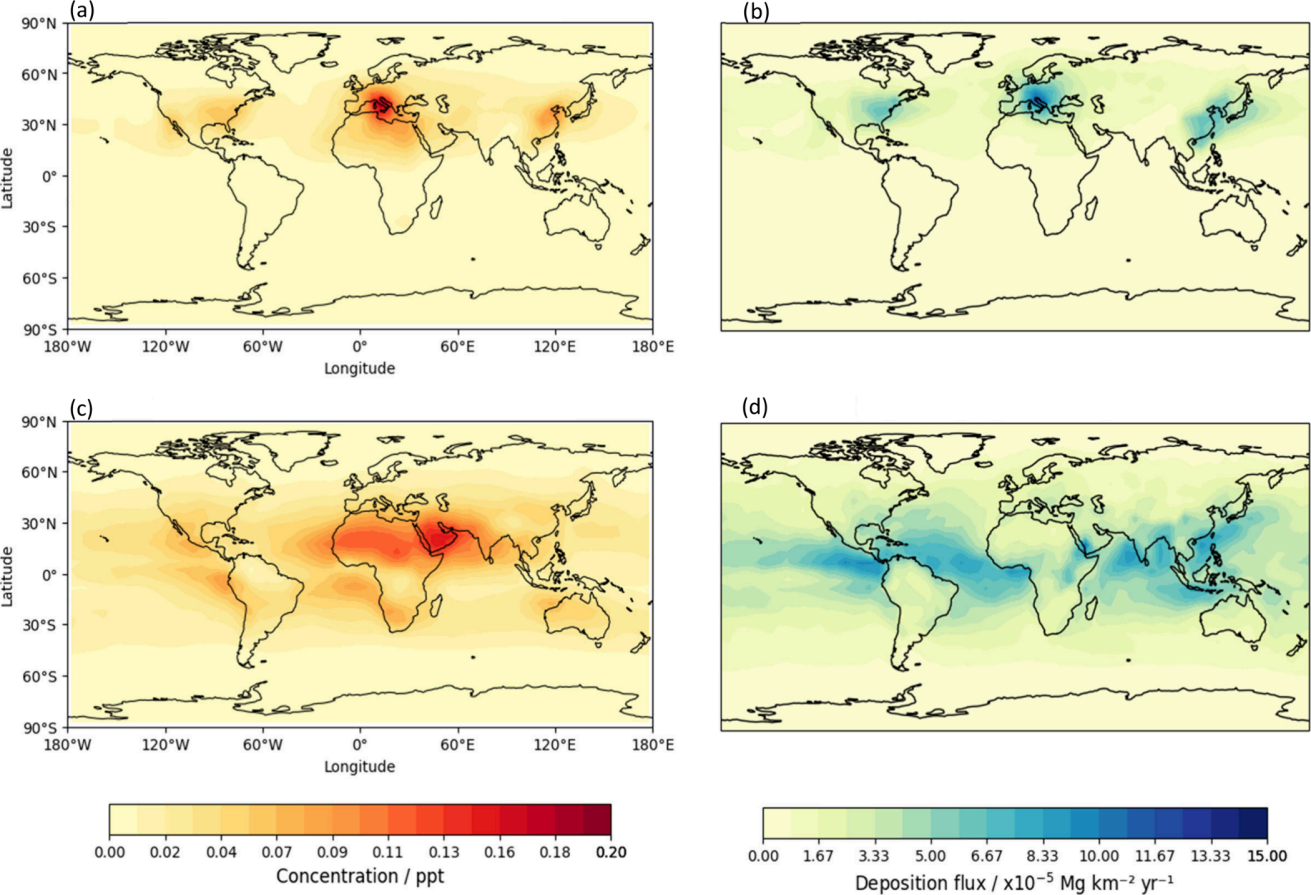
Annual average surface distribution of TFA (a,c) and the average annual total deposition of TFA (b,d) predicted under the HFO-1234yf and HFC-134a_HY scenarios, respectively.

Given the lower TFA yield of 7% in the HFC-134a_LY simulation, we see much lower TFA surface concentrations in this simulation when compared with the HFC-134a_HY scenario. It should be noted that variation in yields between the two HFC-134a scenarios leads to a linear response in TFA generation and deposition. While there has been no further experimental constraint on the true yield of TFA from HFC-134a, a recent study reported a modeled yield of 9%.^43^ If accurate, this would suggest that the true contribution of HFC-134a to TFA generation is more similar to the HFC-134a_LY scenario in this work. This in turn would suggest that TFA generation from HFO-1234yf may be rivalling HFC-134a TFA production globally (∼75%). However, in the absence of further experimental data, we must consider the entire range of uncertainty and will focus on comparison with the HFC-134a_HY scenario as a conservative estimate of the comparative influence of HFO-1234yf and HFC-134a on TFA generation and deposition.

The most significant concern relating to TFA is contamination of inland environmental aqueous phases, of which deposition of atmospherically generated TFA is understood to be a major contributor. Because of the high solubility of TFA, wet deposition has typically been expected to dominate over dry deposition. This is seen in the results of our simulations where wet deposition accounts for around 88–92% of total deposition, with differences driven by the spatial variations of TFA distribution between simulations. Our modeled partial lifetime of 3.5–4.7 days for wet deposition is in reasonable agreement with previous studies (3–15 days).^17,36,55,56^ A recent measurement study has suggested that dry deposition may have a greater, and more variable, contribution to TFA atmospheric loss than previously thought.^55^ It is therefore likely that the dry deposition velocities we utilize are an underestimate. Indeed, our model yields a longer partial lifetime of TFA with respect to dry deposition than the literature (33–43 days compared with 6–20 days).^43,57^ However, in the absence of any recently reported values, they remain the best estimates at present. Additionally, recent reports demonstrate that uncertainty in parameters used to define deposition has a limited influence on deposition rates of TFA.^42,43^

In Figure 1b,d, annually averaged total TFA deposition is presented for each of the scenarios. We see that atmospheric degradation of HFO-1234yf results in more localized TFA deposition, close to source regions and with higher maxima, when compared with HFC-134a_HY. Spatial distributions of TFA deposition are driven by a combination of imposed emission distribution, meteorological input data, and derived oxidant field, all of which are often model-specific thereby making intermodel comparison challenging.

In Europe, modeled HFO-1234yf degradation results in TFA deposition up to 3.6 times higher than that caused by HFC-134a_HY (Figure 2). This increases to a factor of 10.3 when considering the HFC-134a_LY scenario (Figure S8). This result implies a greater contribution to TFA deposition in this area from HFO-1234yf use than from HFC-134a use. Grid cells with the most significant enhancements fall over Italy, parts of Austria, Germany, Switzerland, and France, in reasonable agreement with the location of peak TFA deposition fluxes reported by Henne et al.^9^ Globally, many model grid cells are predicted to see higher TFA deposition in the HFO-1234yf scenario than in the HFC-134a_HY scenario (with a significant increase in number seen when considering the HFC-134a_LY scenario, Figure S8). Similarly, we model such enhancements in parts of North America and East Asia, which warrant further investigation, particularly in the view of recent studies suggesting comparable and greater TFA deposition in these regions, respectively, compared to Europe.^52,58^

**Figure 2:**
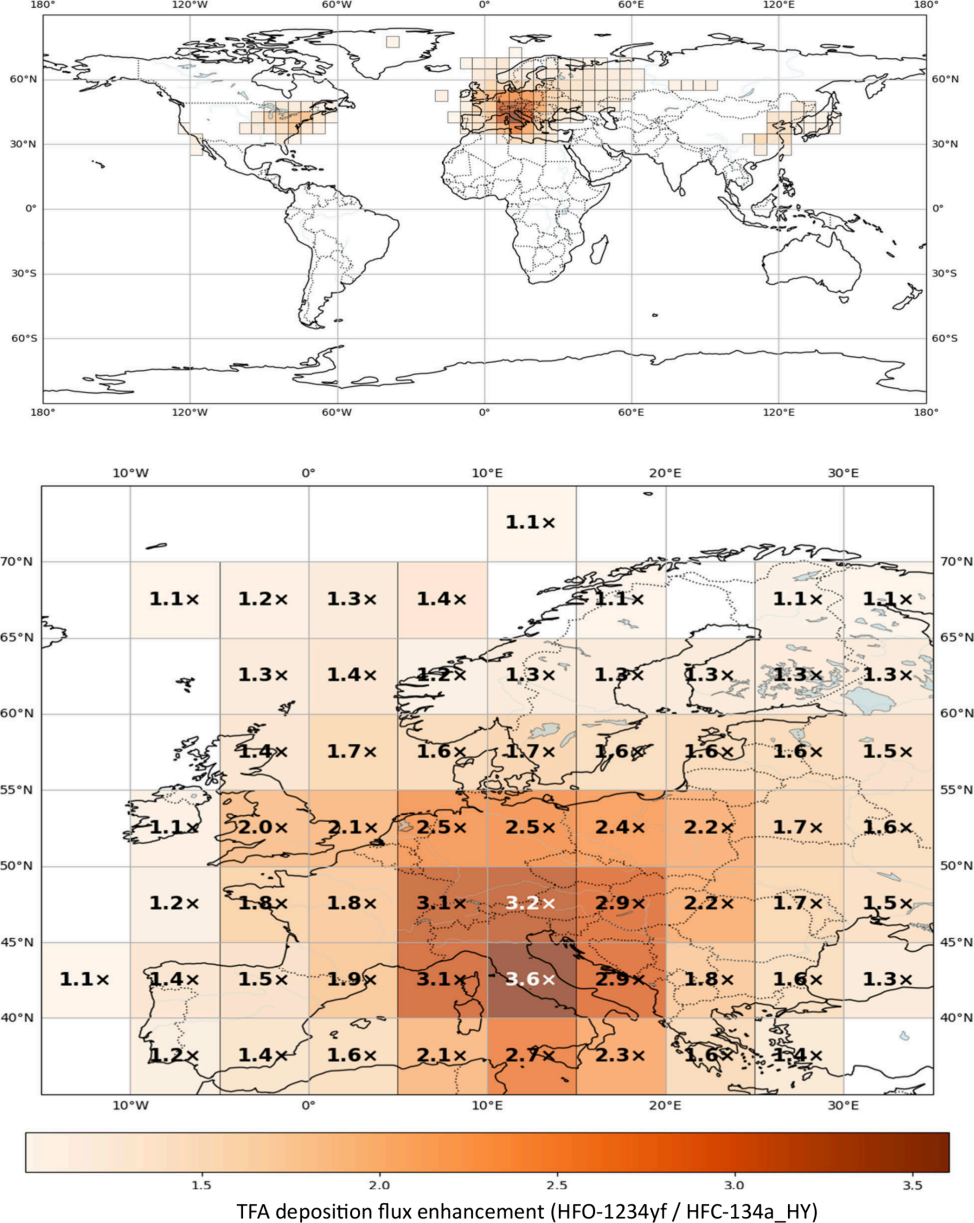
Plots showing the TFA deposition enhancement globally (top row) and over Europe (bottom row) when considering the HFO-1234yf scenario compared to the HFC-134a_HY scenario.

This investigation represents a significant update when compared with our previous work^36^ as, due to the availability of recent estimates for TFA precursor emissions along with atmospheric measurement data to validate model performance, we are now able to simulate a near-present-day scenario relating to atmospheric TFA generation and deposition. In doing so, we find that TFA generation and deposition in Europe are now likely to see a larger contribution from HFO-1234yf degradation than from HFC-134a degradation.

This is predicted to be the case even when we conservatively assume the upper limit of the TFA yield (20%) for HFC-134a. When the lower limit of the TFA yield of HFC-134a is assumed (7%), the comparative impact of HFO-1234yf is expected to be markedly larger. It is vital that the uncertainty in the TFA yield estimate for HFC-134a be reduced to better understand the comparative impacts of HFC-134a and its replacement, HFO-1234yf, on the TFA generation and deposition.

The anticipated transition from HFC-134a to HFO-1234yf is still in its early stages, with emissions of HFC-134a still far exceeding those of HFO-1234yf. As this transition progresses globally and HFO-1234yf emissions increase, its impact on TFA generation and deposition will continue to rise. It is vital that we monitor and understand the full extent of the environmental impacts of source gases, such that informed decisions can be made in industrial and political settings.

Further uncertainty in the magnitude and spatial distribution of HFO-1234yf emissions presents significant barriers to better constraining the contribution of each source gas to TFA. This work highlights a need for improved regional emission magnitude and distribution estimates for important TFA precursors so that their impact on TFA generation and environmental contamination can be better understood.

## Supplementary Material



## Notes

Emissions distribution of each source gas (HFC-134a and HFO-1234yf) utilized in the model, comparison of monthly mean modeled concentrations of the source gas compared with measurements taken from the AGAGE network, comparison of modeled wet and total deposition fluxes with those reported by Persaud et al. in Toronto, Canada, heatmaps visualizing the extent of agreement between our modeled TFA deposition compared with that reported by Henne et al., annual average surface distribution of TFA and total TFA deposition under the HFC-134a_LY scenario, plots showing modeled TFA deposition enhancement in the HFO-1234yf scenario compared with the HFC-134a_LY scenario, and description of how comparisons with reported deposition fluxes were carried out and a brief discussion of the limitations and implications (PDF)
