# Supplementary material for: TFA Generation and Deposition over Europe May Currently See a Greater Influence from HFO-1234yf than HFC-134a
Source: Environ Sci Technol Lett. 2026 Jun 15;13(7):938–45. doi: 10.1021/acs.estlett.6c00356 (PMC13374373; doi:10.1021/acs.estlett.6c00356)
Supplement: Supplementary file 1 [file ez6c00356_si_001.pdf]

TFA generation and deposition over Europe may currently see a greater influence from HFO-1234yf than HFC-134a

Rayne Holland[1*], Ben Adam[1], M Anwar H Khan[1], Dickon Young[1], Simon O'Doherty[1], Kieran M. Stanley[1], Matthew Rigby[1], Dudley E. Shallcross[1,2*]

[1] School of Chemistry, University of Bristol, Cantock's Close, Bristol BS8 1TS, U.K.

[2] Department of Chemistry, University of the Western Cape, Robert Sobukwe Road, Bellville 7535, South Africa

* Corresponding author emails: Rayne Holland (rayne.holland@bristol.ac.uk), Dudley Shallcross (d.e.shallcross@bristol.ac.uk)

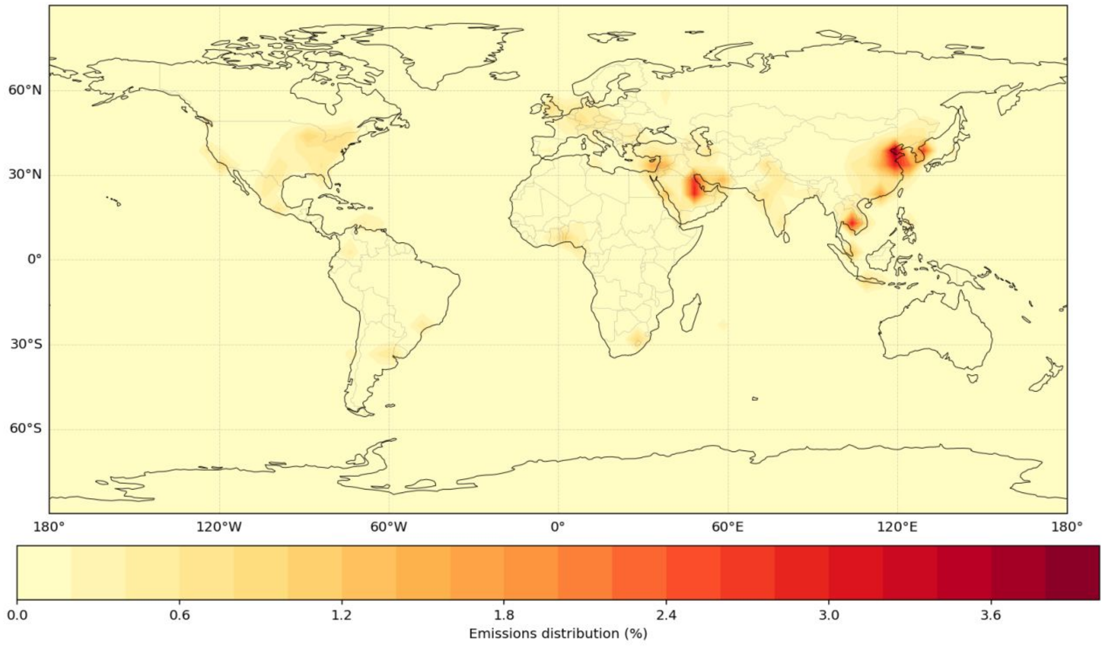

Figure S1: Emission distribution extracted from EDGAR v8[1] utilised for HFC-134a.

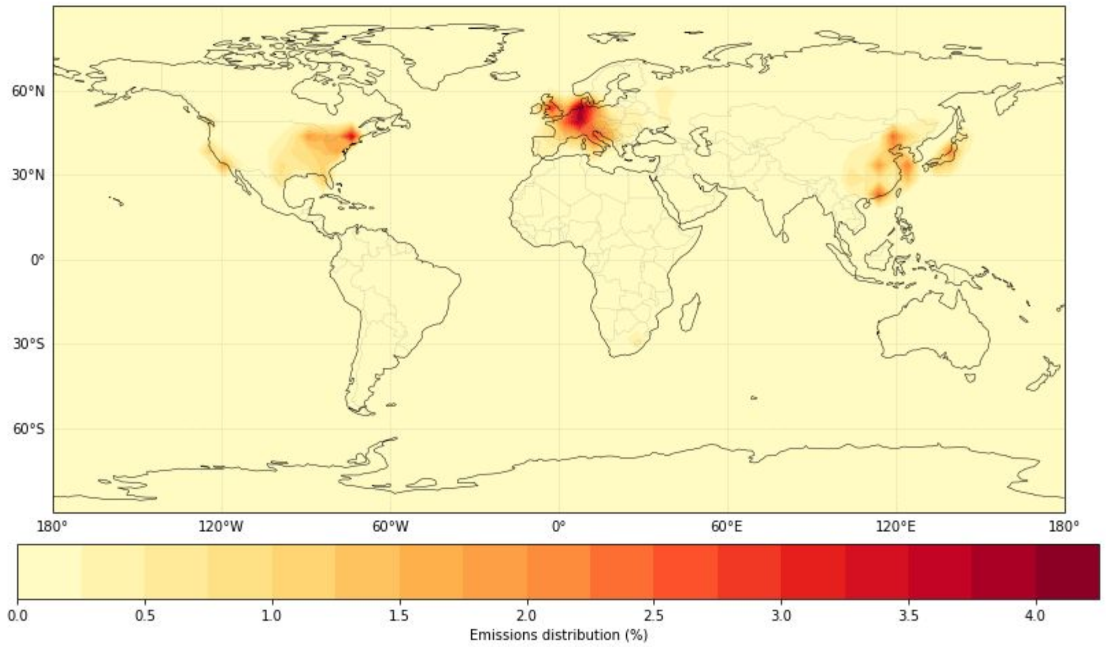

Figure S2: Emission distribution as described in Holland et al.[2] utilised for HFO-1234yf.

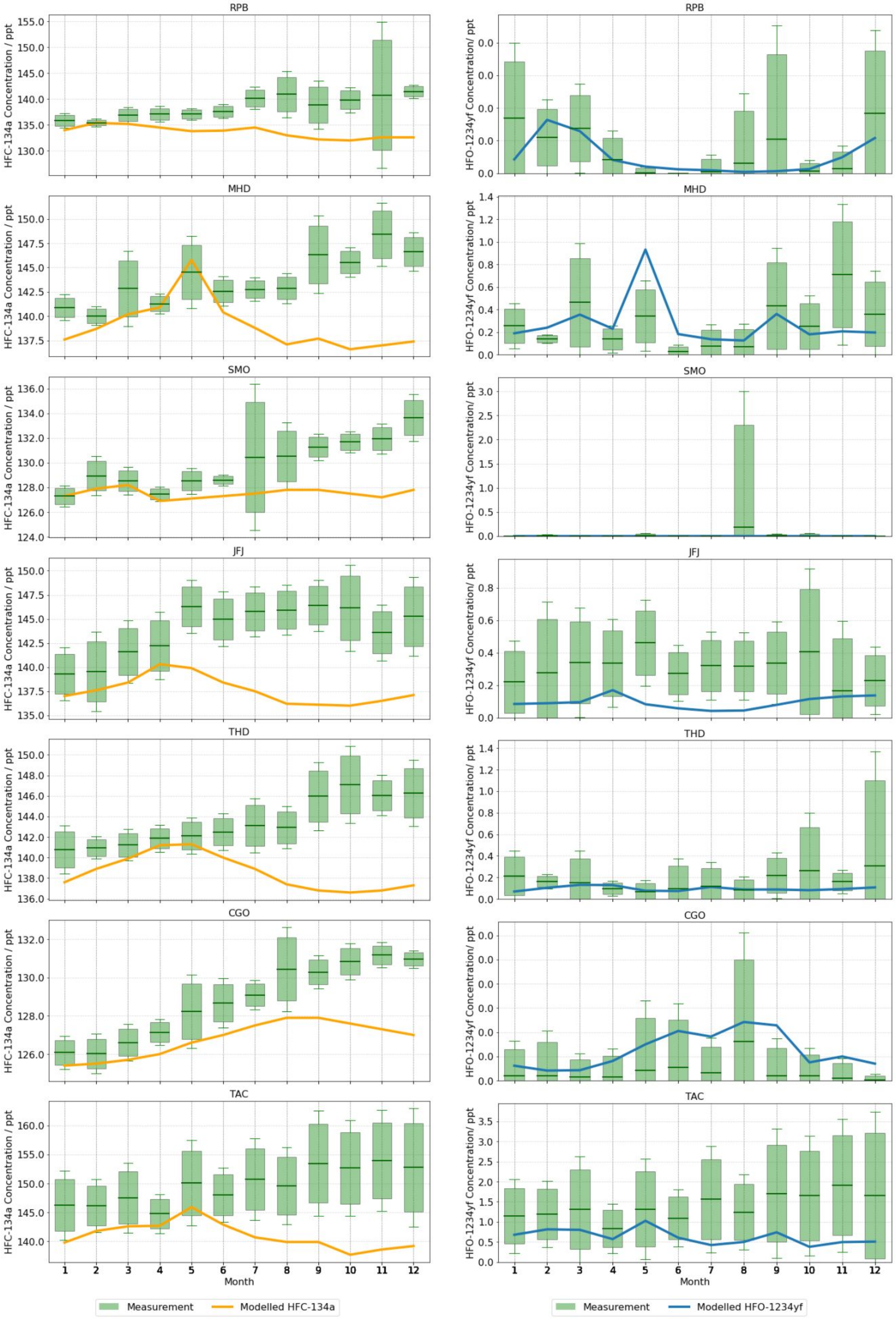

Figure S3: Comparison of monthly mean modelled HFC-134a and HFO-1234yf atmospheric mole fractions with measurements taken via the AGAGE network for the

Ragged Point (RPB), Mace Head (MHD), Cape Matatula (SMO), Jungfraujoch (JFJ), Trinidad Head (THD), Cape Grim (CGO) and Tacolneston (TAC) sites for 2024 (chosen due to completeness of measurement record being much better than 2023). Chosen sites recorded at least one measurement of HFC-134a and HFO-1234yf for each month. Site information can be found at https://www-air.larc.nasa.gov/missions/agage/stations/. HFC-134a data taken from Prinn et al.[1] and HFO-1234yf data taken from Vollmer et al.[3]

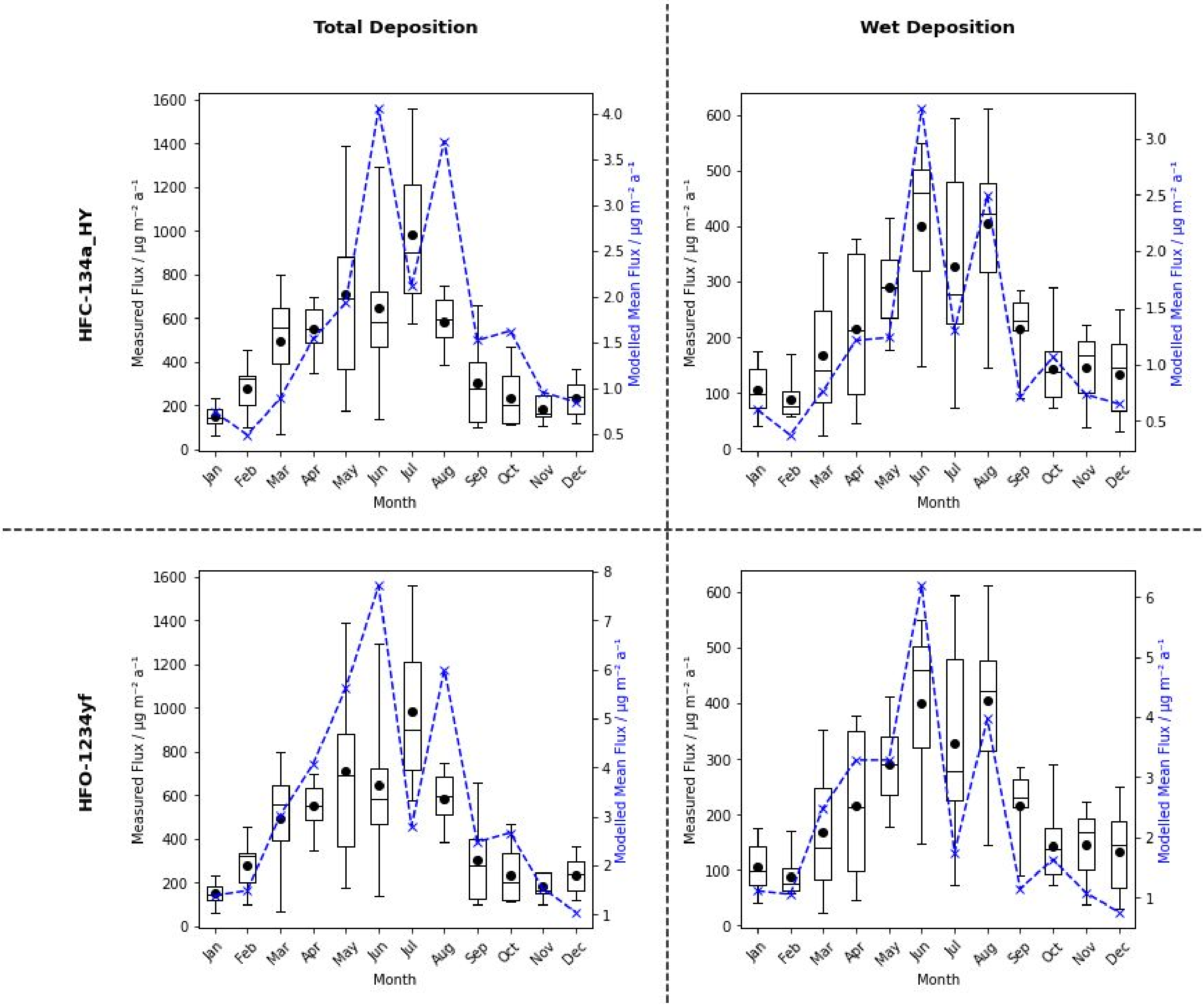

Figure S4: Comparison of modelled total and wet deposition fluxes for TFA (line and right hand axis) in the HFC-134a_HY and HFO-1234yf scenarios with measurement data taken from Persaud et al (box plot, lefthand axis).[4] Box whiskers extend to the full data range, with the mean indicated by a filled circle and the median by a horizontal black line. See Text S1 for discussion.

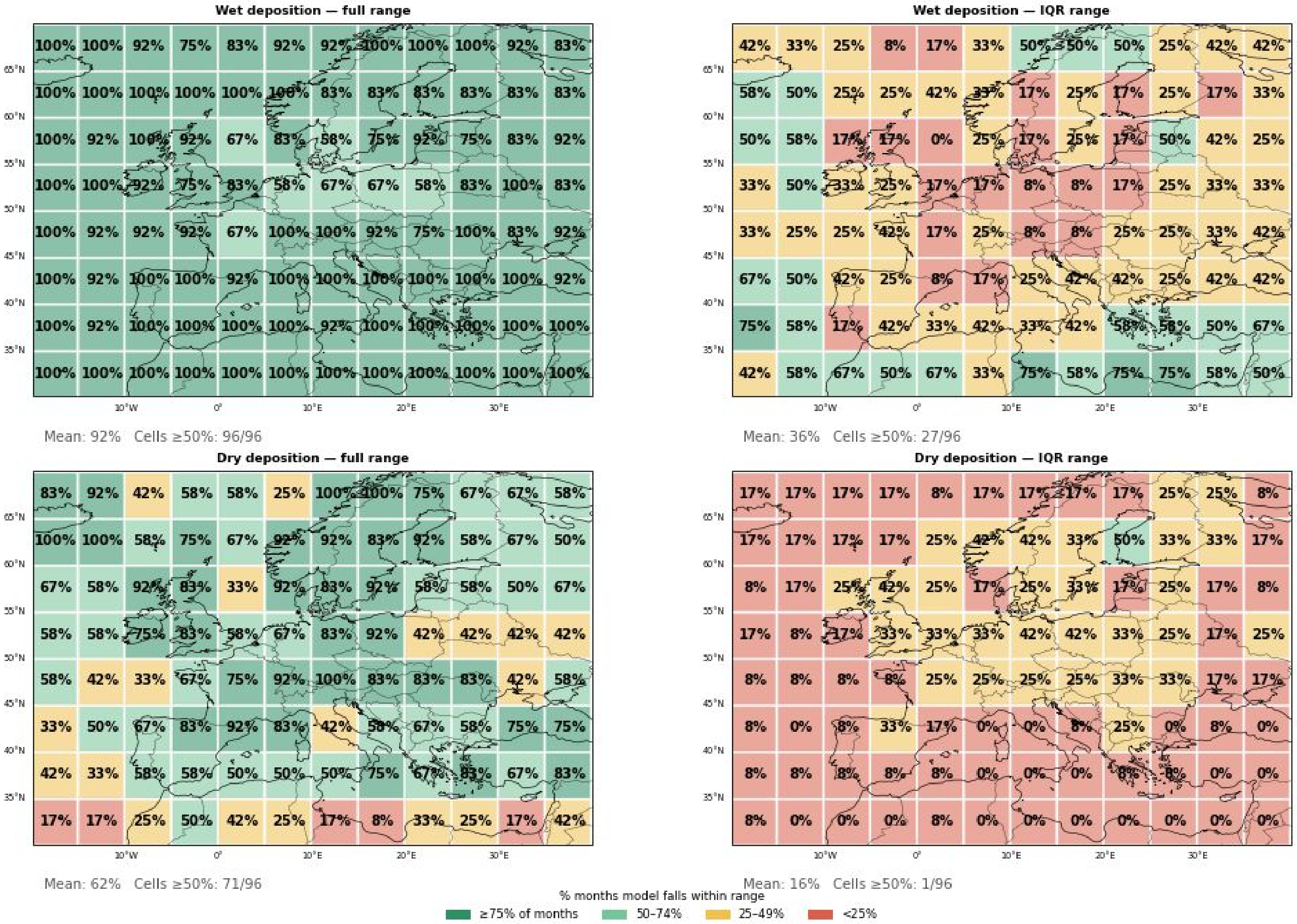

Figure S5: Heatmaps visualising the extent of agreement between our simulated monthly TFA deposition fluxes from HFO-1234yf (from scenario 'HFO-1234yf'), and model results reported by Henne et al.[5] The lefthand column shows the percentage of our modelled deposition values that fell within the full range of data reported by Henne et al. whilst the righthand column shows the percentage of our modelled values which fell within the interquartile range. See Text S1 for method and discussion.

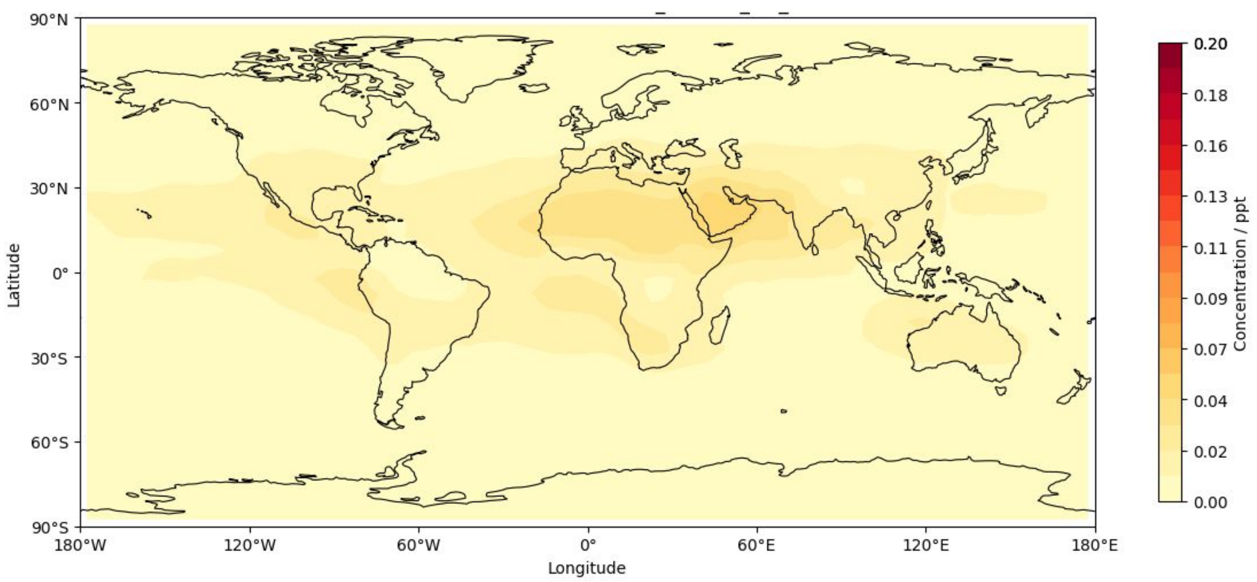

Figure S6: Annual average surface distribution of TFA predicted under the HFC-134a_LY scenario.

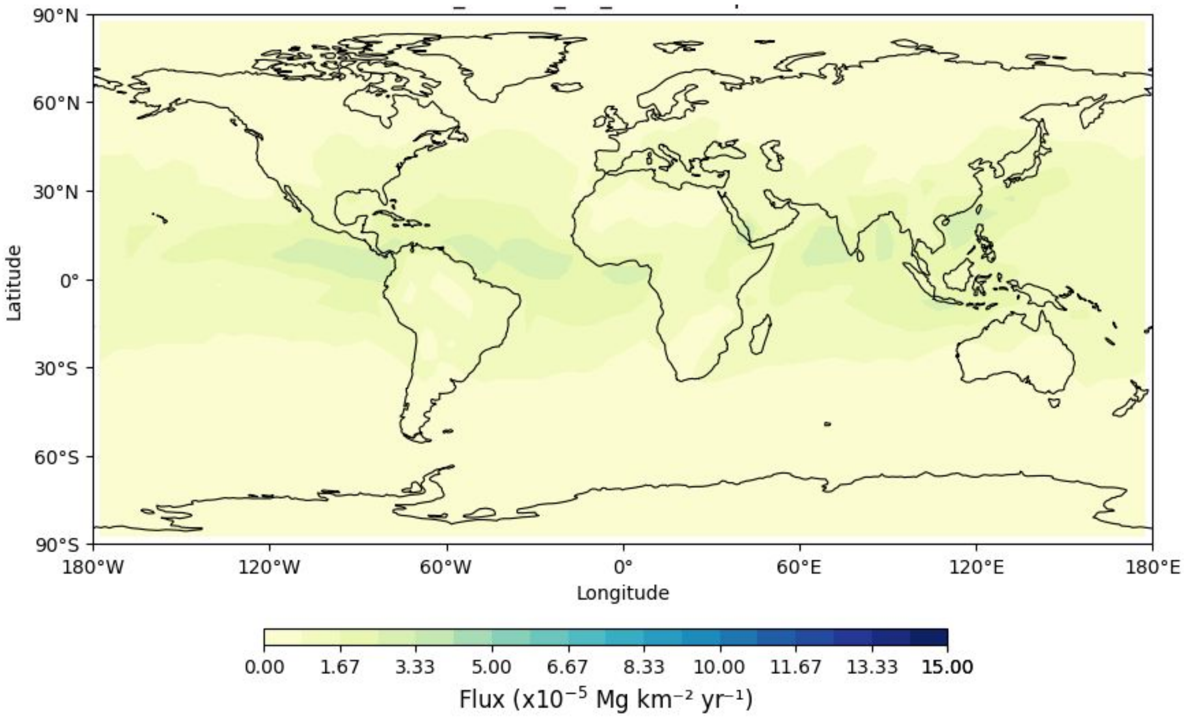

Figure S7: Annual average total deposition of TFA predicted under HFC-134a_LY scenario.

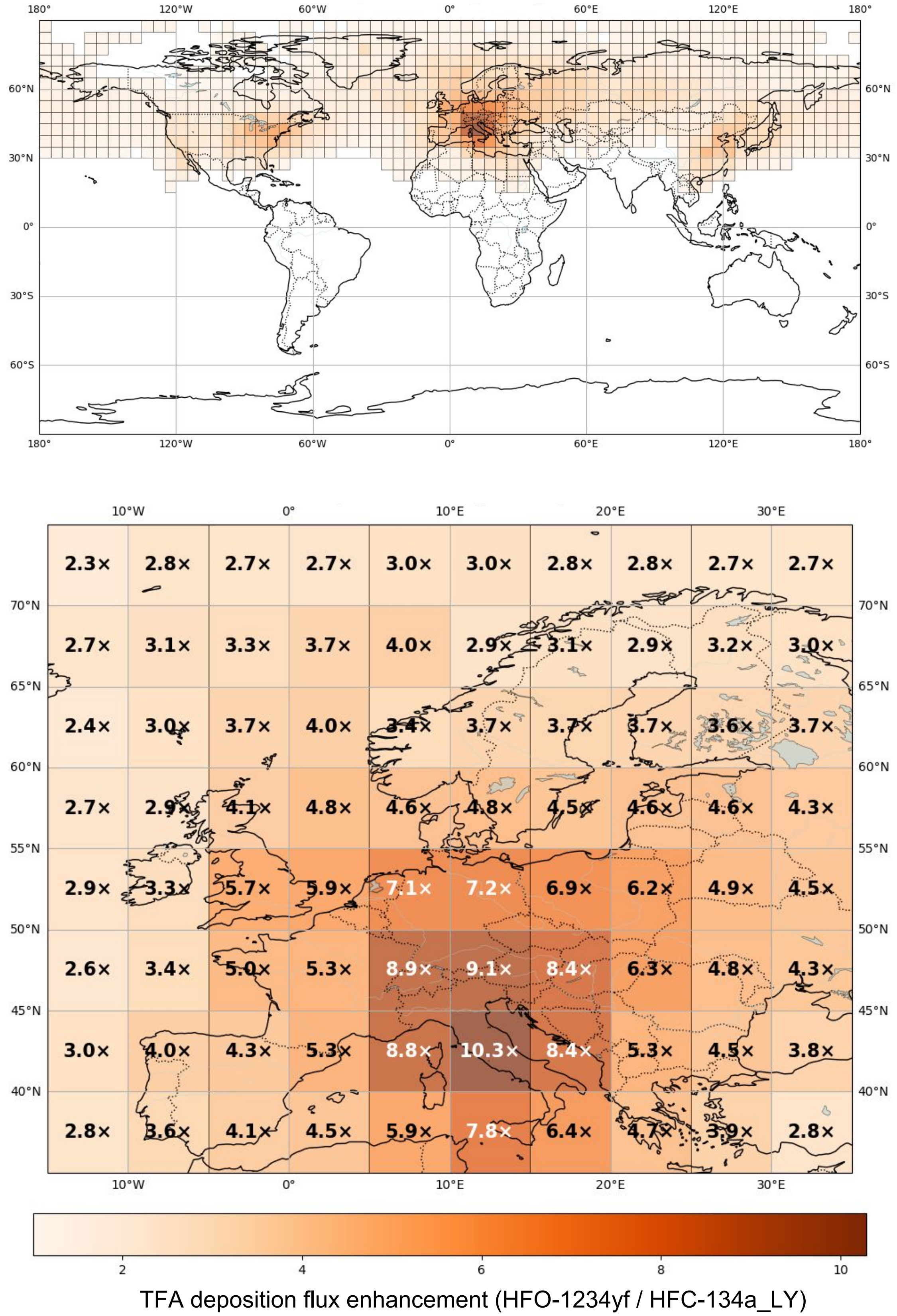

Figure S8: Plots showing the TFA deposition enhancement globally (top row) and over Europe (bottom row) when considering HFO-1234yf scenario compared to the HFC-134a_LY scenario.

Text S1

In Figures S4 and S5 we compare our modelled TFA deposition with recent measurement and modelling studies. Firstly, we compared the seasonality of our modelled TFA deposition to measurements made in Toronto, Canada.[4] While the magnitude of the modelled TFA deposition was significantly lower than in that study, due to our consideration of only a single TFA precursors in each scenario, the seasonality of the measurements was well reproduced in all scenarios (Figure S4).

Similarly, when compared with a recent modelling study looking at European TFA deposition from the breakdown of HFO-1234yf[5], reasonable agreement is seen (Figure S5). Here, we compared our simulated monthly wet and dry deposition fluxes with those reported by Henne et al. Since our model operates on a coarser spatial grid than that of Henne et al., each of our grid cells encompassed multiple deposition flux values from their dataset. We aggregated these values and calculated the minimum, maximum and interquartile range against which we compared our single deposition flux for that grid cell.

For monthly mean wet deposition fluxes of TFA, 92% of our values fall within the range reported and 36% fall within the interquartile range. Where results are not within the range reported, they are primarily underpredicted for wet deposition. For dry deposition, agreement reduces to 62% and 16%, respectively, and differences tend towards overprediction. Overall, we believe this demonstrates our model is able to reasonably represent the complete deposition of TFA.

References

(1) Prinn, R.; Weiss, R.; Arduini, J.; Choi, H.; Engel, A.; Fraser, P.; Ganesan, A.; Harth, C.; Hermansen, O.; Kim, J.; et al. The dataset of in-situ measurements of chemically and radiatively important atmospheric gases from the Advanced Global Atmospheric Gas Experiment (AGAGE) and affiliated stations. Version 20251230 ed.; NASA Langley Research Center (LaRC) Data Host Facility (DHF): 2025.
(2) Holland, R.; Khan, M. A. H.; Driscoll, I.; Chhantyal-Pun, R.; Derwent, R. G.; Taatjes, C. A.; Orr-Ewing, A. J.; Percival, C. J.; Shallcross, D. E. Investigation of the Production of Trifluoroacetic Acid from Two Halocarbons, HFC-134a and HFO-1234yf and Its Fates Using a Global Three-Dimensional Chemical Transport Model. *ACS Earth and Space Chemistry* **2021**, *5* (4), 849-857. DOI: 10.1021/acsearthspacechem.0c00355.
(3) Vollmer, M. K.; Pitt, J. R.; Young, D.; Henne, S.; Mitrevski, B.; Mühle, J.; Ganesan, A.; Arduini, J.; Manning, A. J.; Wagenhäuser, T.; et al. Global Observations and European emissions of the halogenated olefins HFO-1234yf, HFO-1234ze(E), and HCFO-1233zd(E) from the AGAGE (Advanced Global

Atmospheric Gases Experiment) network. *EGUsphere* **2025**, *2025*, 1-46. DOI: 10.5194/egusphere-2025-4824.
(4) Persaud, D.; Joudan, S.; VandenBoer, T. C.; Young, C. J. Atmospheric Removal of Trifluoroacetic Acid by Dry and Wet Deposition: A Multiyear Analysis in Toronto. *Environmental Science & Technology Letters* **2026**, *13* (2), 261-267. DOI: 10.1021/acs.estlett.5c01100.
(5) Henne, S.; Storck, F. R.; Wöhrnschimmel, H.; Leuenberger, M.; Vollmer, M. K.; Reimann, S. Trifluoroacetate (TFA) in precipitation and surface waters in Switzerland: trends, source attribution, and budget. *Atmospheric Chemistry and Physics* **2025**, *25* (23), 18157-18186. DOI: 10.5194/acp-25-18157-2025.
